# Conversations about alcohol in healthcare – cross-sectional surveys in the Netherlands and Sweden

**DOI:** 10.1186/s12889-020-8367-8

**Published:** 2020-03-04

**Authors:** Latifa Abidi, Per Nilsen, Nadine Karlsson, Janna Skagerström, Amy O’Donnell

**Affiliations:** 1 Department of Health Promotion, Maastricht University, Maastricht, Limburg Netherlands; 2 Department of Health, Medicine and Caring Sciences, Linköping University, Linköping, Sweden; 3 Research and Development Unit of Region Östergötland, Region Östergötland, Linköping, Sweden; 4 Population Health Sciences Institute, Newcastle University, Newcastle upon Tyne, NE2 4AX UK

**Keywords:** Alcohol, Brief intervention, Healthcare, Prevention, Implementation

## Abstract

**Background:**

This study evaluated and compared the extent, duration, contents, experiences and effects of alcohol conversations in healthcare in the Netherlands and Sweden in 2017.

**Methods:**

Survey data in the Netherlands and Sweden were collected through an online web panel. Subjects were 2996 participants (response rate: 50.8%) in Sweden and 2173 (response rate: 82.2%) in the Netherlands. Data was collected on socio-demographics, alcohol consumption, healthcare visits in the past 12 months, number of alcohol conversations, and characteristics of alcohol conversations (duration, contents, experience, effects).

**Results:**

Results showed that Swedish respondents were more likely to have had alcohol conversations (OR = 1.99; 95%CI = 1.64–2.41; *p* = < 0.001) compared to Dutch respondents. In Sweden, alcohol conversations were more often perceived as routine (*p* = < 0.001), were longer (*p* = < 0.001), and more often contained verbal information about alcohol’s health effects (*p* = 0.007) or written information (*p* = 0.001) than in the Netherlands. In Sweden, 40+ year-olds were less likely to report a positive effect compared to the youngest respondents. In the Netherlands, men, sick-listed respondents, and risky drinkers, and in Sweden those that reported “other” occupational status such as parental leave, were more likely to have had alcohol conversations.

**Conclusions:**

The results suggest that alcohol conversations are more common in healthcare practice in Sweden than in the Netherlands. However, positive effects of alcohol conversations were less likely to be reported among older respondents in Sweden. Our results indicate that alcohol preventative work should be improved in both countries, with more focus on risky drinkers and the content of the conversations in Sweden, and expanding alcohol screening in the Netherlands.

## Background

Alcohol consumption is causally related to over 200 diseases, injuries, physical and mental health conditions [30, 41, 47], and is one of the most important risk factors for ill-health and premature death [21, 32]. Risky drinking is also associated with various adverse societal consequences, such as domestic violence, child abuse, lost productivity in the workplace, and crime [29, 49].

Addressing the harms associated with alcohol consumption remains an important public health challenge for governments worldwide [56]. A range of measures can help reduce risky drinking, including behavioral and pharmacological treatments delivered in healthcare settings [15, 44]. Brief interventions (BI) are conversations between patients and healthcare workers that aim to reduce alcohol consumption and related harm in risky drinkers who are not actively seeking help for their drinking. BIs may include feedback on alcohol use, information about harms, and advice on how to reduce alcohol consumption [25]. There is a particularly robust evidence base showing that BI delivered in primary healthcare result in significant reductions in alcohol consumption [6, 7, 25, 26, 50], as well as savings to healthcare services [4, 45]. However, widespread implementation of BI in primary healthcare has been difficult to achieve [24, 54].

Healthcare practitioners report experiencing various barriers to asking patients about their alcohol use including: a perceived lack of knowledge about the early symptoms of excessive alcohol use [48]; insufficient time and resources [24]; and the impact of their own drinking practices [10, 18]. These barriers may explain why BI delivery in global healthcare systems remains limited. One study in the Netherlands showed that alcohol use is the least discussed lifestyle topic during primary healthcare consultations [40]. A further survey conducted in England found that less than 10% of those who drink excessively reported having received advice on their alcohol consumption in primary healthcare [9].

BI research has thus far paid relatively little attention to patients’ perspectives on alcohol consultations in healthcare, with the exception of a few, mostly qualitative, studies [22, 28, 31, 51]. A previous study conducted in 2010 in Sweden [37, 38] found that one-fifth of the population who had visited healthcare in the previous 12 months had experienced one or more conversations about alcohol. More than one in five excessive drinkers that were surveyed said the conversation led to reduced alcohol consumption, and most reported that they found the conversation informative, providing valuable knowledge about the impact of alcohol on health [27, 37, 38]. No studies have been conducted in the Netherlands that have investigated the duration, contents, experiences and effects of alcohol-related consultations in routine healthcare practice from the viewpoint of patients.

There are a number of social, political and economic similarities between Sweden and the Netherlands. Both are high-income countries [58] and have similar rates of alcohol consumption in the population [59]. Sweden and the Netherlands also report similar rates of risky alcohol consumption: 17% in 2016 16,8% in 2015 respectively (although it should be noted that there are differences in how consumption is measured in each country) ([12, 17, 35, 36];). Both countries have also implemented policy measures aimed at increasing BI delivery in healthcare in recent years. National guidelines were introduced in Sweden in 2011 to encourage improved delivery of lifestyle advice by practitioners in routine healthcare, including alcohol consumption [52]. In 2014, government reforms to the Dutch mental healthcare system included increased support for the prevention of problematic alcohol use through the introduction of mental health practice nurses in general practices [55, 57]. To date, however, we have limited insight into the impact of these policy changes on the implementation of alcohol BI in Dutch or Swedish healthcare systems.. Responding to this knowledge gap, this study aimed to investigate the extent to which alcohol is currently addressed in patient conversations in routine healthcare in Sweden and the Netherlands, and the duration, contents, experiences and effects of such conversations, using population-based cross-sectional surveys.

In doing so, we sought to address the following research questions:

1. To what extent has the population in the Netherlands and Sweden visited healthcare and had a conversation about alcohol in the past 12 months, and does this vary between different categories of drinkers?
2. How extensive are these alcohol conversations (duration), what do they include (contents), how are they experienced (experience), and what is their impact (effects), and does this vary between different categories of drinkers and/or between countries?
3. What characterizes those individuals who are most likely to have had a conversation about alcohol in healthcare in the Netherlands and Sweden?

## Methods

### Study population and design

This study has a cross-sectional design, comprising surveys conducted in Sweden [27] and the Netherlands. The Netherlands sample consisted of 2645 panel members, representative of the Dutch population. Survey data were collected from the LISS (Longitudinal Internet Studies for the Social sciences; http://www.lissdata.nl) panel, administered by CentERdata (Tilburg University, The Netherlands). Questionnaires were sent to participants between April and May 2017. All participants gave informed consent to complete the questionnaire. The Swedish sample consisted of 5900 nationally representative panel members [37, 38]. Survey data were collected by means of a web panel conducted by EnkätFabriken, an organisation which specializes in survey research (http://www.enkatfabriken.se). Participants were sent an electronic survey questionnaire between August and September 2017.

### Questionnaire

The original questionnaire was in Swedish [27, 37, 38]. To ensure accuracy in the Dutch translated version, two native speakers backwards-forwards translated the questionnaire from Swedish to Dutch and vice versa. Any discrepancies in the translated questionnaire were discussed until agreement was reached. The questionnaire itself included 14 questions. Five questions collected socio-demographic background data on the respondent’s age, gender, marital status, occupational status, and level of education. Three questions were based on the Alcohol Use Disorder Identification Test – Consumption (AUDIT-C) instrument [11]: [1] How often have you consumed alcohol in the past 12 months? (never; less often than once a month; approximately once a month; two to three times per month; one to two times per week; three to four times per week; daily or almost daily), [2] How many standard glasses do you usually drink when you drink alcohol? [3] How often do you drink 5 standard glasses or more if you are a man, and 4 standard glasses or more if you are a woman, on one occasion, for example during an evening (never; less often than once a month; approximately once a month; two-three times a month; one-two times per week; three-four times per week; daily or almost daily).

On the basis of responses to the AUDIT-C questions, three alcohol consumption categories were constructed: abstainers; moderate drinkers; and risky drinkers. Abstainers were defined in both countries as respondents that reported not drinking in the past 12 months. Definitions of moderate and risky drinkers were country-specific. According to the Swedish guidelines [3, 52], moderate drinking is defined as consuming: up to 14 drinks (168 g) per week or five drinks (60 g) on a single occasion for men; and up to nine drinks (108 g) per week or four drinks (48 g) on a single occasion for women, where one standard Swedish drink contains 12 g of pure alcohol. Drinking above this level is defined as risky drinking. According to the Dutch Health Council, moderate drinking is defined as consuming: up to 14 drinks (140 g) per week for men or five drinks (50 g) on a single occasion for men; and up to seven drinks (70 g) per week or four drinks (40 g) on a single occasion for women [8, 19], where a standard drink contains 10 g of pure alcohol. Drinking above this level is defined as risky drinking.

One question was used to assess whether the respondent had visited healthcare during the previous 12 months (response options: yes, once; yes, more than once; no). Those who responded “yes” to this question were also asked: Have you talked about your alcohol consumption at any consultation in healthcare in the past 12 months? (response options: yes, once; yes, more than once; no). For those respondents who had discussed their drinking with a healthcare provider, the following four questions were used to measure the *duration, contents, experience* and *effects* of the conversation:

*Duration*: How long was the latest conversation about alcohol? Possible responses were: less than 1 min; 1–4 min; 5–10 min; more than 10 min.

*Contents:* What did the conversation include? Possible responses were: verbal information about the impact of alcohol on health (Yes/No); questions about how much alcohol I usually drink (Yes/No); questions about whether I would like to reduce my drinking (Yes/No); practical advice on how to reduce my drinking (Yes/No); written information about alcohol (Yes/No).

*Experience:* What was your experience of the conversation? Possible responses were: it provided valuable knowledge; it was informative; it was routine; it felt awkward; it was frustrating; and it felt judgmental. Answers to these statements were given by respondents on a four-item Likert scale, with answer options ranging from “do not agree” to “agree completely”.

*Effects:* Did the conversation affect you in any way? Possible responses were: it had no effect at all; it made me think about my drinking; it gave me a better understanding of alcohol’s health risks; it led to an increase in my drinking; It led to a reduction in my drinking; and it made me think about a friend’s drinking. Answers to these statements were given by respondents on a four-item Likert scale, with answer options ranging from “do not agree” to “agree completely”.

### Analyses

Sample characteristics were analysed using descriptive statistics. Two-way tables of characteristics of conversations about alcohol in healthcare versus country were produced, and analysed using the chi-squared test. The Dutch and Swedish datasets were merged.

An initial multivariable logistic regression model was performed to investigate associations between the determinants age, gender, educational level, occupation, marital status, drinking categories, number of healthcare visits in the past 12 months, country, and the primary outcome variable ‘having had a conversation about alcohol in the past 12 months (yes versus no)’. Interactions between country and these determinants were tested in the logistic regression model using the Wald test of interaction.

A second multivariable logistic regression model was performed to investigate associations between the same determinants and a secondary outcome variable based on three possible responses to the item ‘having had a conversation about alcohol in the past 12 months and reporting a positive effect’, namely: 1) made me consider my drinking; 2) gave me a better understanding of alcohol’s health risks; or 3) led to reduction of my drinking. This variable was constructed to allow for analysis of determinants of the reported positive effects of the conversation about alcohol.

Sensitivity analyses were conducted to investigate differences in grams of alcohol in standard glasses in both countries (10 g of alcohol per standard drink in the Netherlands versus 12 g of alcohol in Sweden). A multivariable logistic regression model was performed in which the converted “drinking categories” variable (standardized to weekly consumption in grams, with 12 g of alcohol per standard drink in both countries) and all other determinants were included as predictors. As such, in the sensitivity analyses, a “standard drink” in the Netherlands and Sweden contained an equal amount of alcohol.

Data were analyzed using the statistical software package for Windows SPSS 24. Results were considered statistically significant at *p <* 0.05 using two-tailed tests.

## Results

The response rate of individuals who answered the complete survey questionnaire was 50.8% (*n* = 2996) in Sweden and 82.2% (*n* = 2173) in the Netherlands. Table 1 shows that the countries differed significantly on all sample characteristics.

**Table 1:** Sample characteristics

Variables	Country		*p*-value
	Sweden	Netherlands	
Sex	**(*n*** **= 2996)**	**(*n*** **= 2173)**	0.013
Man	1501 (50.1%)	1015 (46.7%)	
Women	1495 (49.9%)	1158 (53.3%)	
Age	**(*n*** **= 3000)**	**(*n*** **= 2173)**	< 0.001
Mean age (SD)	40.6 (13.4%)	52.6 (17.9)	
< 29 years	851 (28.4%)	316 (14.5%)	
30–39 years	604 (20.1%)	259 (11.9%)	
40–49 years	630 (21.0%)	296 (13.6%)	
50–59 years	591 (19.7%)	390 (17.9%)	
60+ years	324 (10.8%)	912 (42.0%)	
Education	**(*n*** **= 3000)**	**(*n*** **= 2173)**	< 0.001
Basic	141 (4.7%)	94 (4.3%)	
Secondary school	1392 (46.4%)	689 (31.7%)	
Post-secondary education	1467 (48.9%)	1311 (60.3%)	
Other	0 (0.0%)	79 (3.6%)	
Occupation	**(*n*** **= 2999)**	**(*n*** **= 2173)**	< 0.0010
Employed	2227 (74.2%)	1025 (47.2%)	
Student	359 (12.0%)	179 (8.2%)	
Unemployed	98 (3.3%)	64 (2.9%)	
Sick-listed	82 (2.7%)	72 (3.3%)	
Retired	142 (4.7%)	567 (26.1%)	
Other	91 (3.0%)	266 (12.2%)	
Marital status	**(*n*** **= 3000)**	**(*n*** **= 2173)**	< 0.001
Married/living together	1897 (63.2%)	1486 (68.4%)	
Single or living apart	1103 (36.8%)	619 (28.5%)	
Other	0 (0.0%)	68 (3.1%)	
Healthcare visits in the last 12 months	**(*n*** **= 3000)**	**(*n*** **= 2171)**	< 0.001
2 or more visits	1113 (37.1%)	1095 (50.4%)	
1 visit	930 (31.0%)	482 (22.2%)	
No visit	957 (31.9%)	594 (27.3%)	
Conversations about alcohol in healthcare in the last 12 months	**(*n*** **= 2043)**	**(*n*** **= 1577)**	< 0.001
2 or more conversations	120 (5.9%)	99 (6.3%)	
1 conversation	416 (20.4%)	198 (12.6)	
No conversation	1507 (73.8%)	1280 (81.2%)	
Conversations about alcohol in healthcare in the last 12 months & reported a positive effect	**(*n*** **= 423)**	**(*n*** **= 263)**	< 0.001
1, 2 or more conversations and effect	322 (76.1%)	150 (57.0%)	
1, 2 or more conversations and no effect	101 (23.9%)	113 (43.0%)	
Drinking categories	**(*n*** **= 2996)**	**(*n*** **= 2172)**	< 0.001
Abstainers	284 (9.5%)	352 (16.2%)	
Moderate drinkers	1865 (62.2%)	1174 (54.0%)	
Risky drinkers	847 (28.2%)	646 (29.7%)	

Table 2 shows data about alcohol conversations in healthcare in Sweden and the Netherlands. In Sweden, more conversations about alcohol lasted 1–4 min or longer, while in the Netherlands, conversations about alcohol mostly lasted less than 1 min (*p* = < 0.001). There was also a difference in the content of the conversation, with verbal information about alcohol’s influence on health (*p* = 0.007) and written information about alcohol (*p* = 0.001) provided more often in Sweden than in the Netherlands. The experience of the conversation about alcohol was perceived more often as routine in Sweden than in the Netherlands (*p* = < 0.001), but it was experienced similarly regarding the other measures (i.e. awkward, irritating, judgmental, informative or providing knowledge).

**Table 2:** Characteristics of conversation about alcohol in healthcare

Variables	Sweden				Netherlands			Total	*p*-value
	Abstainers	Moderate drinkers	Risky drinkers	Total	Abstainers	Moderate drinkers	Risky drinkers		
	N (%)	N (%)	N (%)	N (%)	N (%)	N (%)	N (%)	N (%)	
**Healthcare visits in the past 12 months**	**(*n*** **= 284)**	**(*n*** **= 1865)**	**(*n*** **= 847)**	**(*n*** **= 2996)**	**(*n*** **= 351)**	**(*n*** **= 1174)**	**(*n*** **= 646)**	**(*n*** **= 2171)**	< **0.001**
2 or more visits	122 (43.0)	694 (37.2)	295 (34.8)	1111 (37.1)	189 (53.8)	611 (52.0)	295 (45.7)	1095 (50.4)	
1 visit	88 (31.0)	582 (31.2)	259 (30.6)	929 (31.0)	68 (19.4)	270 (23.0)	144 (22.3)	482 (22.2)	
No visit	74 (26.1)	589 (31.6)	293 (34.6)	956 (31.9)	94 (26.8)	293 (25.0)	207 (32.0)	594 (27.3)	
**Conversation about alcohol in healthcare in the past 12 months**	**(*n*** **= 210)**	**(*n*** **= 1276)**	**(*n*** **= 554)**	**(*n*** **= 2040)**	**(*n*** **= 257)**	**(*n*** **= 881)**	**(*n*** **= 439)**	**(*n*** **= 1577)**	< **0.001**
2 or more conversations	16 (7.6)	63 (4.9)	40 (7.2)	119 (5.8)	18 (7.0)	48 (5.4)	33 (7.5)	99 (6.3)	
1 conversation	40 (19.0)	260 (20.4)	115 (20.8)	415 (20.3)	21 (8.2)	114 (12.9)	63 (14.4)	198 (12.6)	
No conversation	154 (73.3)	953 (74.7)	399 (72.0)	1506 (73.8)	218 (84.8)	719 (81.6)	343 (78.1)	1280 (81.2)	
**Duration of conversation about alcohol**	**(*n*** **= 56)**	**(*n*** **= 323)**	**(*n*** **= 155)**	**(*n*** **= 534)**	**(*n*** **= 39)**	**(*n*** **= 162)**	**(*n*** **= 96)**	**(*n*** **= 297)**	< **0.001**
< 1 min	45 (80.4)	207 (64.1)	72 (46.5)	324 (60.7)	37 (94.9)	132 (81.5)	59 (61.5)	228 (76.7)	
1–4 min	7 (12.5)	94 (29.1)	58 (37.4)	159 (29.8)	2 (5.1)	28 (17.3)	22 (22.9)	52 (17.5)	
5–10 min	4 (7.1)	18 (5.6)	20 (12.9)	42 (7.8)	0 (0.0)	2 (1.2)	11 (11.5)	13 (4.4)	
> 10 min	0 (0.0)	4 (1.2)	5 (3.2)	9 (1.7)	0 (0.0)	0 (0.0)	4 (4.1)	4 (1.3)	
Contents of conversation about alcohol (use dichotomized variables) *(affirmative answers)*									
Verbal information about alcohol’s influence on health	12 (21.4)	80 (24.8)	51 (32.9)	143 (26.9)	13 (33.3)	22 (13.6)	20 (20.8)	55 (18.5)	**0.007**
Questions about my alcohol consumption	49 (87.5)	290 (89.8)	130 (83.9)	469 (87.9)	32 (82.1)	146 (90.1)	87 (90.6)	265 (89.2)	0.560
Questions about my willingness to reduce consumption	0 (0.0)	22 (6.8)	28 (18.1)	50 (9.3)	2 (5.1)	7 (4.3)	21 (21.9)	30 (10.1)	0.717
Advice on how to reduce my consumption	0 (0.0)	12 (3.7)	20 (12.9)	32 (6.0)	2 (5.1)	3 (1.9)	9 (9.4)	14 (4.7)	0.447
Written information about alcohol	3 (5.4)	25 (7.7)	15 (9.7)	43 (8.0)	2 (5.1)	0 (0.0)	5 (5.2)	7 (2.4)	**0.001**
Experiences of conversation about alcohol *(agreed completely or to a large degree)*									
Provided valuable knowledge	18 (32.1)	87 (26.9)	42 (27.1)	147 (27.6)	15 (38.5)	39 (24.1)	25 (26.0)	79 (26.6)	0.143
It was informative	22 (39.3)	99 (30.6)	47 (30.4)	168 (31.5)	18 (46.2)	63 (38.9)	33 (34.4)	114 (38.4)	0.082
It was routine	51 (91.1)	302 (93.5)	135 (87.1)	488 (91.5)	29 (74.4)	125 (77.1)	60 (62.5)	214 (72.0)	< **0.001**
It felt awkward	3 (5.4)	11 (3.4)	19 (12.2)	33 (6.2)	4 (10.2)	6 (3.7)	8 (8.3)	18 (6.1)	0.786
It was irritating	4 (7.2)	15 (4.6)	13 (8.4)	32 (5.9)	2 (5.2)	2 (1.2)	16 (16.7)	10 (3.4)	0.119
It was offensive	1 (1.8)	8 (2.4)	11 (7.1)	20 (3.8)	X	X	X	X	
It felt judgmental	1 (1.8)	8 (2.4)	11 (7.1)	20 (3.8)	2 (5.2)	4 (2.4)	3 (3.1)	9 (3.0)	0.769
Effects of conversation about alcohol *(agreed completely or to a large degree)*									
Had no effect at all	44 (78.6)	242 (74.9)	92 (59.3)	378 (70.5**)**	31 (79.5)	136 (83.9)	60 (62.5)	227 (76.4)	0.217
Made me consider my drinking	2 (3.6)	23 (6.8)	27 (17.5)	52 (9.5)	3 (7.7)	15 (9.3)	21 (21.9)	39 (13.1)	0.335
Gave me a better understanding of alcohol’s health risks	6 (10.7)	34 (10.5)	25 (16.1)	65 (12.1)	13 (33.4)	47 (29.0)	33 (34.4)	93 (31.3)	< **0.001**
Led to increase in my drinking	1 (1.8)	6 (1.9)	8 (5.2)	15 (2.8)	2 (5.2)	3 (1.8)	2 (2.1)	7 (2.3)	0.492
Led to reduction of my drinking	5 (8.9)	24 (7.4)	21 (13.5)	50 (9.4)	4 (10.3)	20 (12.4)	17 (17.7)	41 (13.8)	0.054
Made me think about a friend’s drinking	8 (14.3)	37 (11.5)	21 (13.5)	66 (12.3)	7 (18.0)	12 (7.5)	12 (12.5)	31 (10.4)	< **0.001**

A higher proportion of respondents in the Netherlands reported that the conversation gave them a better understanding of alcohol’s health risks (31.3% vs. 12.1%; *p* = < 0.001), and a higher proportion of respondents in Sweden reported that the conversation made them think about a friend’s drinking (10.4% vs. 12.3%; *p* = < 0.001).

In the multivariable logistic regression model in Table 3 (model 1), women had a lower odds ratio of having had a conversation in healthcare compared to men (OR = 0.81; 95%CI = 0.69–0.96; *p* = 0.015). Respondents in Sweden were almost twice as likely to have had a conversation about alcohol in the past 12 months compared to respondents from the Netherlands (OR = 1.99; 95%CI = 1.64–2.41; *p* = < 0.001). Respondents that were long term sick-listed and those listed as “other” (on parental leave; volunteering) were more likely to have had an alcohol conversation in healthcare compared to those who were employed (OR = 1.51; 95% CI = 1.09–2.08; *P* = 0.012; OR = 1.64; 95%CI = 1.11–2.40; *p* = 0.012, respectively). In the Netherlands, risky drinkers, and those who reported two or more visits to healthcare, had a higher odds ratio of having had an alcohol conversation (OR = 1.41; 95%CI = 1.06–1.87; *p* = 0.016; OR = 2.68; 95% CI = 2.23–3.22; *p* = < 0.001). None of the interactions between the predictors and country were statistically significant.

**Table 3:** Logistic regression model of having had a conversation about alcohol in healthcare in the past 12 months overall (model 1), in Sweden (model 2), in the Netherlands (model 3) in function of determinants

Variables	Overall model				Sweden				Netherlands			
	N	OR^a^	95%CI	*p*-value	N	OR^a^	95%CI	*p*-value	N	OR^a^	95%CI	*p*-value
Sex												
Male	1621	1			928	1			693	1		
Female	1996	0.81	(0.69–0.96)	0.015*	1112	0.84	(0.70–1.07)	0.179	884	0.75	(0.57–0.99)	0.040*
Age												
16–29 years	750	1			542	1			208	1		
30–39 years	582	1.07	(0.80–1.43)	0.641	418	1.07	(0.77–1.48)	0.683	164	1.04	(0.53–2.07)	0.902
40–49 years	612	0.86	(0.64–1.17)	0.344	422	0.94	(0.67–1.31)	0.701	190	0.78	(0.39–1.57)	0.481
50–59 years	691	0.91	(0.68–1.22)	0.534	415	0.96	(0.68–1.34)	0.806	276	0.92	(0.48–1.76)	0.804
60+ years	982	1.19	(0.86–1.64)	0.278	243	1.26	(0.84–1.90)	0.257	739	1.31	(0.68–2.53)	0.414
Education												
Primary education	171	1			106	1			65	1		
Secondary	1461	1.05	(0.71–1.55)	0.793	943	1.15	(0.70–1.87)	0.581	518	0.85	(0.44–1.65)	0.638
University	1934	1.07	(0.73–1.59)	0.727	991	1.11	(0.68–1.82)	0.677	943	0.88	(0.45–1.70)	0.704
Other	51	1.41	(0.66–3.02)	0.382	0	N.A	N.A	N.A.	51	1.23	(0.49–3.04)	0.661
Occupation												
Employed	2144	1			1470	1			674	1		
Student	349	1.11	(0.79–1.55)	0.555	230	1.20	(0.82–1.75)	0.353	119	0.93	(0.43–1.98)	0.844
Unemployed	116	1.37	(0.89–2.12)	0.151	71	1.41	(0.84–2.37)	0.198	45	1.24	(0.56–2.74)	0.593
Sick-listed	140	1.64	(1.11–2.40)	0.012*	79	1.31	(0.80–2.16)	0.280	61	2.06	(1.12–3.79)	0.021*
Retired	587	1.01	(0.75–1.37)	0.936	116	0.99	(0.62–1.62)	0.999	471	0.83	(0.53–1.29)	0.408
Other	281	1.51	(1.09–2.08)	0.012*	74	2.57	(1.54–4.27)	0.000*	207	1.03	(0.64–1.66)	0.889
Marital status												
Married/living together	2351	1			1294	1			1057	1		
Single or living apart	1215	1	(0.84–1.19)	0.97	746	0.98	(0.78–1.22)	0.830	469	1.12	(0.84–1.48)	0.441
Other	51	0.92	(0.45–1.88)	0.818	0	N.A	N.A	N.A	51	0.92	(0.45–1.91)	0.833
Drinking categories												
Abstainers	467	1			210	1			257	1		
Moderate drinkers	2151	1.18	(0.91–1.52)	0.22	1267	1.08	(0.76–1.54)	0.658	881	1.35	(0.91–2.00)	0.141
Risky drinkers	999	1.41	(1.06–1.87)	0.016*	554	1.25	(0.86–1.84)	0.245	439	1.74	(1.13–2.68)	0.012*
Healthcare visits												
1 visit	1411	1			929	1			482	1		
2 or more visits	2206	2.68	(2.23–3.22)	< 0.001*	1111	2.49	(2.00–3.09)	< 0.001*	1095	3.29	(2.30–4.70)	< 0.001*
Country												
Netherlands	1577	1										
Sweden	2040	1.99	(1.64–2.41)	< 0.001*	N.A	N.A	N.A	N.A	N.A	N.A	N.A	N.A

*Abbreviations: OR* odds ratio; *CI* confidence interval; ^a^ORs are adjusted for age, sex, educational level, occupation, marital status, drinking categories, healthcare visits in the past 12 months, and country; * = significant at *P*-value ≤.05. Interaction between predictors and country were non-significant and removed from the model = country*sex: *P* = 0.416; country*age: *P* = 0.975; country*education: *P* = 0.765; country*occupation: *P* = 0.101; country*marital status: *P* = 0.459; country*drinking categories: *P* = 0.537; country*number of visits to healthcare: *P* = 0.191

In the multivariable logistic regression model in Table 4, only the interaction between country*age was significant (*P* = 0.031). In Sweden, those aged 40+ years were less likely to report a positive effect of their alcohol conversation compared to the youngest age category. The stratified analyses of both countries are presented in Table 4. Stratified analyses show that in both countries, women had a lower odds ratio of both having had a conversation in healthcare and having reported a positive effect, compared to men (SW: OR = 0.41; 95%CI = 0.28–0.59; *p* = 0.001; NL: OR = 0.32; 95%CI = 0.17–0.57; *p* = 0.001).

**Table 4:** Logistic regression model of having had a conversation about alcohol in healthcare in the past 12 months and having reported a positive effect in function of determinants

Variables	Overall model				Sweden				Netherlands			
	N	OR^a^	95%CI	*p*-value	N	OR^a^	95%CI	*p*-value	N	OR^a^	95%CI	*p*-value
Sex												
Male	310	1			183	1			127	1		
Female	376	0.41	(0.28–0.59)	< 0.001*	240	0.41	(0.24–0.69)	< 0.001*	136	0.32	(0.17–0.57)	< 0.001*
Age												
16–29 years	127	1			101	1			26	1		
30–39 years	128	0.73	(0.38–1.40)	0.351	102	0.58	(0.28–1.19)	0.138	27	1.48	(0.26–8.40)	0.655
40–49 years	105	0.40	(0.19–0.83)	0.015*	81	0.35	(0.15–0.79)	0.012*	24	0.53	(0.07–3.72)	0.520
50–59 years	120	0.69	(0.35–1.33)	0.265	78	0.36	(0.16–0.81)	0.014*	42	2.52	(0.49–12.90)	0.267
60+ years	206	0.85	(0.42–1.69)	0.638	62	0.29	(0.11–0.76)	0.012*	144	3.64	(0.71–18.69)	0.121
Education												
Primary education	35	1			21	1			14	1		
Secondary school	281	1.16	(0.53–2.55)	0.714	197	0.86	(0.30–2.50)	0.783	84	1.48	(0.41–5.26)	0.546
University	362	0.72	(0.33–1.59)	0.422	205	0.68	(0.23–2.00)	0.490	157	0.69	(0.20–2.40)	0.565
Other	8	0.66	(0.12–3.73)	0.642	N.A	N.A	N.A	N.A	8	0.41	(0.04–3.87)	0.438
Occupation												
Employed	387	1			289	1			98	1		
Student	57	0.77	(0.35–1.67)	0.504	43	0.74	(0.32–1.75)	0.497	14	0.75	(0.11–5.14)	0.773
Unemployed	28	1.02	(0.41–2.55)	0.969	22	0.62	(0.20–1.93)	0.408	6	3.39	(0.35–32.56)	0.289
Sick-listed	40	0.77	(0.33–1.75)	0.528	21	0.30	(0.06–1.43)	0.132	19	0.96	(0.27–3.43)	0.949
Retired	110	1.20	(0.65–2.24)	0.555	22	1.57	(0.50–4.89)	0.438	88	0.80	(0.32–2.03)	0.638
Other	64	0.76	(0.38–1.52)	0.439	26	0.45	(0.12–1.65)	0.228	38	0.63	(0.23–1.72)	0.373
Marital status												
Married/living together	452	1			281	1			171	1		
Single or living apart	226	1.02	(0.70–1.50)	0.897	142	1.01	(0.60–1.70)	0.962	84	0.98	(0.53–1.79)	0.939
Other	8	0.54	(0.11–2.54)	0.438	0	N.A	N.A	N.A	8	0.53	(0.09–2.92)	0.463
Drinking categories												
Abstainers	80	1			46	1			34	1		
Moderate drinkers	412	0.78	(0.44–1.40)	0.411	265	1.17	(0.49–2.79)	0.725	147	0.59	(0.24–1.41)	0.237
Risky drinkers	194	1.60	(0.87–2.95)	0.131	112	2.35	(0.94–5.88)	0.067	82	1.61	(0.63–4.09)	0.321
Healthcare visits in the past 12 months												
1 visit	157	1			122	1			35	1		
2 or more visits	529	1.14	(0.73–1.77)	0.564	301	1.27	(0.73–2.20)	0.398	228	1.34	(0.57–3.11)	0.502
Country												
Netherlands	263	1			N.A	N.A	N.A		N.A	N.A	N.A	
Sweden	423	0.43	(0.28–0.66)	< 0.001*	N.A	N.A	N.A	N.A	N.A	N.A	N.A	N.A

*Abbreviations: OR* odds ratio, *CI* confidence interval; ^a^ORs are adjusted for age, sex, educational level, occupation, marital status, drinking categories, healthcare visits in the past 12 months, and country; * = significant at *P*-value ≤.05, N.A: not applicable

Interaction between predictors and country = country*age: *P* = 0.031; country*sex: *P* = 0.518; country*education: *P* = 0.423; country*occupation: *P* = 0.498; country*marital status: *P* = 0.929; country*drinking categories: *P* = 0.494; country*number of visits to healthcare: *P* = 0.919

The sensitivity analyses for the first outcome variable (having had a conversation about alcohol in the past 12 months) and for the second outcome variable (having had a conversation about alcohol in healthcare in the past 12 months and having reported a positive effect) revealed similar results to those described in Table 3 and Table 4 (see Supplementary Tables).

## Discussion

We found that respondents in Sweden were almost twice as likely to have had a conversation about alcohol in the past 12 months compared to respondents from the Netherlands. Nine out of ten Swedish respondents stated that the conversation on alcohol was routine, compared to seven out of ten in the Netherlands. In Sweden, alcohol-related conversations were more likely to be longer in duration, and to contain both verbal and written information about alcohol’s influence on health, compared to the Netherlands. Interestingly, whilst alcohol conversations in the Netherlands were less likely to contain verbal or written information about the impact of excessive alcohol consumption on health, respondents were more likely than those in Sweden to report improved understanding of alcohol-related risks as a result of the conversation.

These findings might indicate that alcohol BI are more common in Swedish healthcare compared to Dutch healthcare, potentially due to the sustained preventative alcohol initiatives implemented by the Swedish government over recent decades. The 2004 Risk Drinking Project aimed to make questions about alcohol consumption a routine part of Swedish healthcare. Educational activities undertaken as part of this project reached several sections of the Swedish healthcare system such as primary care, antenatal care, and occupational care [37, 38]. A repeated cross-sectional study carried out in one Swedish county showed that the prevalence of being asked or advised on alcohol consumption in healthcare increased from 15% in 2004 (before the Risk Drinking Project was launched) to 25% in 2008 and 33% in 2012 [5, 23, 34]. A recent study comparing alcohol conversations in Swedish healthcare practice in 2010 and 2017 also found evidence that alcohol advice had become more embedded in healthcare practice over time [27]. This contrasts with the situation in the Netherlands, where one study using video-recordings of medical consultations found that alcohol use was the least discussed lifestyle topic in primary healthcare [40].

A higher proportion of respondents in the Netherlands reported that the conversation gave them a better understanding of alcohol-related health risks compared to those in Sweden. One explanation might be that the level of information provided to patients about the impact of risky drinking is already high in Sweden. Additionally, risky drinkers in the Netherlands were more likely to have had an alcohol-related conversation compared to moderate drinkers or abstainers. It is therefore possible that these individuals would have an increased likelihood of benefiting from discussing their drinking with a health professional [2].

Our finding that risky drinkers in the Netherlands were more likely to have had an alcohol-related conversation than other categories of drinkers also reflects those from a previous UK study, which found that risky drinkers were more likely than abstainers or moderate drinkers to be asked about their alcohol use [33]. In Sweden however, our results echo those from a previous population study which found that risky drinkers did not report alcohol conversations in healthcare more often than abstainers or moderate drinkers [37, 38]. Given that risky drinkers are the target group for alcohol BI, these results suggest a need for a more effective delivery strategy to be implemented in Sweden in the future. The fact that risky drinkers in the Netherlands were more likely to have had an alcohol-related conversation than other categories of drinkers might reflect the more targeted approach to alcohol screening and BI that is recommended in national guidelines [8]. This contrasts with the Swedish guidelines, which recommend alcohol BI delivery to any patient identified as “having an increased risk” [3, 52].

Women in the Netherlands were less likely than men to have had a conversation about alcohol with their healthcare provider. This finding is consistent with other studies in healthcare settings, which suggest that women are less likely than men to be asked about their alcohol consumption [1, 33], potentially as a result of the higher rates of alcohol-related problems found in men [13]. Interestingly, in both the Netherlands and Sweden, women were also less likely to report a positive effect related to the alcohol conversation. This might be due to a lower prevalence of risky drinking among women in general, meaning there is less need for women to consider or reduce their drinking. However it could also reflect the fact that women are more interested in health-related information, and more active in seeking and discussing this information than men [14].

Respondents in Sweden that were listed as “other” under occupation (including those on parental leave) were more likely to have had a conversation about alcohol in healthcare than employed respondents. Our findings are in line with a previous study in Sweden that also showed that those on parental leave were more likely to have had a conversation about alcohol in healthcare than those in employment [37, 38]. In this respect, it is important to note that Sweden has an extensive system of maternity health centres, which is free of charge and easy accessible. Additionally, all pregnant women are screened for alcohol use in Swedish antenatal care, as are many partners.

Participants in Sweden aged 40 or over were less likely to report a positive effect related to the alcohol conversation compared to younger participants. Whilst previous research suggests small but positive effects of alcohol BI in elderly populations [16, 20], there is also evidence that older drinkers are less likely to be supportive of routinely addressing alcohol in healthcare [43], and might be more reluctant to disclose information about their drinking [42]. Such factors may have contributed to more skeptical reporting on the effects of alcohol BI in older respondents in our sample.

Several policy and legislative factors could have contributed to the differences reported here between Sweden and the Netherlands. In particular, despite moving towards a more liberal approach after joining the European Union in 1995, Sweden has more restrictive alcohol policies targeting availability compared to the Netherlands. For example, the Swedish government has a monopoly on alcohol retail (i.e. “Systembolaget”), with regulated opening hours, no offers or sales and a minimum age of 20 years to buy alcohol (18 years in restaurants, clubs, and bars). Furthermore, drinks are taxed more heavily in Sweden than in most other parts of the world [46]. In the Netherlands, although the minimum age to buy alcohol increased from 16 to 18 years as of January 2014, alcoholic beverages are more readily available to consumers, with beer, wine and low alcohol content spirits sold in both grocery stores and licensed liquor stores. As such, Sweden’s more restrictive alcohol policies could have helped normalize lower risk drinking, and have provided additional legitimacy to the need to address excessive alcohol use in healthcare. Indeed, a previous study in Sweden showed that there is considerable support amongst the population for practitioners asking routine questions about alcohol in healthcare [39].

This is the first study to investigate cross-country differences regarding alcohol conversation in healthcare based on large representative samples of the Dutch and Swedish population. The data collected allow a detailed study of the views of the general population in Sweden and the Netherlands than has been obtained in previous patient studies.

However this study also has limitations that must be acknowledged when interpreting the findings. Different methods of recruitment were employed in each country, and the limited response rate in Sweden (50.8% compared to 82.2% in the Netherlands) might have led to selective samples that are also less comparable. Our analyses revealed significant differences in sample characteristics between the countries, which might be related to the response rates. Some of the observed differences between the samples were small, and as we had large sample sizes, even small differences in characteristics might reach significance level. Further, self-report data were used, which may be sensitive to social desirability and recall bias [53]. The questionnaire was relatively short in order to obtain a high response rate. Therefore, certain follow-up questions about the experience of the alcohol conversations that might explain some of the results, or cultural, sociodemographic and occupational variables of healthcare professionals, could not be included. Follow-up questions about how many patients address alcohol themselves and about health status (symptoms, diagnoses, and medications) might also provide further insight and alternative explanations to some of the results. Lastly, the amount of alcohol in a standard drink is not the same in both countries, which could have affected our findings. However, the sensitivity analyses conducted as part of this study suggest this was not the case.

## Conclusion

The results suggest that conversations about alcohol are more common in routine healthcare practice in Sweden than in the Netherlands. However, positive effects of alcohol conversations were less likely to be reported among respondents in Sweden aged 40 or over. Our findings indicate that alcohol preventative work should be improved in both countries, with more focus on risky drinkers and the content of the conversations in Sweden, and by expanding alcohol screening provision in the Netherlands.

## Supplementary information

**Additional file 1.** Sensitivity analyses.

## Data Availability

The datasets used and/or analysed during the current study are available from the corresponding author on reasonable request.

## Abbreviations

AUDIT-C: Alcohol Use Disorder Identification Test-Consumption
BI: Brief Intervention
CI: Confidence Interval
LISS: Longitudinal Internet Studies for the Social sciences
OR: Odds Ratio
