# Supplementary material for: Conversations about alcohol in healthcare – cross-sectional surveys in the Netherlands and Sweden
Source: BMC Public Health. 2020 Mar 4;20:283. doi: 10.1186/s12889-020-8367-8 (PMC7057588; doi:10.1186/s12889-020-8367-8)
Supplement: Supplementary file 1 — Additional file 1. Sensitivity analyses. [file 12889_2020_8367_MOESM1_ESM.docx]

**Supplementary tables – Sensitivity analyses including standardized grams per drink (12 grams)**

**Table 5: Logistic regression model of having had a conversation about alcohol in healthcare in the past 12 months overall (model 1), in Sweden (model 2), in the Netherlands (model 3) in function of determinants**

|  |  |  |  |  |  |  | |  |  |  |  | |  |  |
| --- | --- | --- | --- | --- | --- | --- | --- | --- | --- | --- | --- | --- | --- | --- |
| **Variables** | **Overall model Sweden Netherlands** | | | | | | | | | | | | |  |
|  |  |  |  |  |  |  |  | |  |  |  |  | |  |
|  | **N** | **OR^a^** | **95%CI** | **p-value** | **N** | **OR^a^** | **95%CI** | | **p-value** | **N** | **OR^a^** | **95%CI** | | **p-value** |
|  |  |  |  |  |  |  |  | |  |  |  |  | |  |
| **Sex** |  |  |  |  |  |  |  | |  |  |  |  | |  |
| Male | 1621 | 1 |  |  | 928 | 1 |  | |  | 693 | 1 |  | |  |
| Female | 1996 | 0.81 | (0.69-0.96) | **0.013*** | 1112 | 0.86 | (0.70-1.07) | | 0.179 | 884 | 0.75 | (0.57-0.98) | | **0.038*** |
|  |  |  |  |  |  |  |  | |  |  |  |  | |  |
| **Age** |  |  |  |  |  |  |  | |  |  |  |  | |  |
| 16-29 years | 750 | 1 |  |  | 542 | 1 |  | |  | 208 | 1 |  | |  |
| 30-39 years | 582 | 1.06 | (0.80-1.42) | 0.680 | 418 | 1.07 | (0.77-1.48) | | 0.683 | 164 | 1.01 | (0.51-2.00) | | 0.981 |
| 40-49 years | 612 | 0.85 | (0.63-1.15) | 0.303 | 422 | 0.94 | (0.67-1.31) | | 0.701 | 190 | 0.73 | (0.37-1.46) | | 0.378 |
| 50-59 years | 691 | 0.90 | (0.67-1.21) | 0.491 | 415 | 0.96 | (0.68-1.34) | | 0.806 | 276 | 0.87 | (0.46-1.67) | | 0.686 |
| 60+ years | 982 | 1.18 | (0.85-1.63) | 0.318 | 243 | 1.26 | (0.84-1.90) | | 0.257 | 739 | 1.26 | (0.65-2.42) | | 0.490 |
|  |  |  |  |  |  |  |  | |  |  |  |  | |  |
| **Education** |  |  |  |  |  |  |  | |  |  |  |  | |  |
| Primary education | 171 | 1 |  |  | 106 | 1 |  | |  | 65 | 1 |  | |  |
| Secondary school | 1461 | 1.05 | (0.71-1.55) | 0.800 | 943 | 1.15 | (0.70-1.87) | | 0.581 | 518 | 0.85 | (0.44-1.65) | | 0.634 |
| University | 1934 | 1.07 | (0.72-1.58) | 0.741 | 991 | 1.11 | (0.68-1.82) | | 0.677 | 943 | 0.88 | (0.45-1.69) | | 0.694 |
| Other | 51 | 1.39 | (0.65-2.99) | 0.393 | 0 | N.A | N.A | | N.A | 51 | 1.21 | (0.49-3.01) | | 0.681 |
|  |  |  |  |  |  |  |  | |  |  |  |  | |  |
| **Occupation** |  |  |  |  |  |  |  | |  |  |  |  | |  |
| Employed | 2144 | 1 |  |  | 1470 | 1 |  | |  | 674 | 1 |  | |  |
| Student | 349 | 1.11 | (0.80-1.55) | 0.532 | 230 | 1.20 | (0.82-1.75) | | 0.353 | 119 | 0.93 | (0.44-1.20) | | 0.854 |
| Unemployed | 116 | 1.36 | (0.88-2.10) | 0.159 | 71 | 1.41 | (0.84-2.37) | | 0.198 | 45 | 1.20 | (0.54-2.65) | | 0.650 |
| Sick-listed | 140 | 1.62 | (1.10-2.38) | **0.014*** | 79 | 1.31 | (0.80-2.16) | | 0.280 | 61 | 2.00 | (1.09-3.68) | | **0.026*** |
| Retired | 587 | 1.00 | (0.74-1.36) | 0.989 | 116 | 1.00 | (0.62-1.62) | | 0.999 | 471 | 0.81 | (0.52-1.26) | | 0.352 |
| Other | 281 | 1.49 | (1.08-2.06) | **0.014*** | 74 | 2.57 | (1.54-4.27) | | **0.000*** | 207 | 1.02 | (0.64-1.63) | | 0.936 |
|  |  |  |  |  |  |  |  | |  |  |  |  | |  |
| **Marital status** |  |  |  |  |  |  |  | |  |  |  |  | |  |
| Married/living together | 2351 | 1 |  |  | 1294 | 1 |  | |  | 1057 | 1 |  | |  |
| Single or living apart | 1215 | 1.00 | (0.85-1.20) | 0.946 | 746 | 0.98 | (0.78-1.22) | | 0.830 | 469 | 1.13 | (0.85-1.50) | | 0.399 |
| Other | 51 | 0.92 | (0.45-1.89) | 0.823 | 0 | N.A | N.A | | N.A | 51 | 0.93 | (0.45-1.93) | | 0.845 |
|  |  |  |  |  |  |  |  | |  |  |  |  | |  |
| **Drinking categories** |  |  |  |  |  |  |  | |  |  |  |  | |  |
| **(Adjusted grams=12)** |  |  |  |  |  |  |  | |  |  |  |  | |  |
| Abstainers | 467 | 1 |  |  | 210 | 1 |  | |  | 257 | 1 |  | |  |
| Moderate drinkers | 2388 | 1.21 | (0.93-1.56) | 0.149 | 1267 | 1.08 | (0.76-1.54) | | 0.658 | 1112 | 1.43 | (0.97-2.11) | | 0.072 |
| Risky drinkers | 762 | 1.38 | (1.02-1.85) | **0.033*** | 554 | 1.25 | (0.86-1.84) | | 0.245 | 208 | 1.66 | (1.02-2.71) | | **0..043*** |
|  |  |  |  |  |  |  |  | |  |  |  |  | |  |
| **Healthcare visits** |  |  |  |  |  |  |  | |  |  |  |  | |  |
| 1 visit | 1411 | 1 |  |  | 929 | 1 |  | |  | 482 | 1 |  | |  |
| 2 or more visits | 2206 | 2.68 | (2.23-3.22) | **<0.000*** | 1111 | 2.49 | (2.00-3.09) | | **0.000*** | 1095 | 3.29 | (2.30-4.70) | | **0.000*** |
|  |  |  |  |  |  |  |  | |  |  |  |  | |  |
| **Country** |  |  |  |  |  |  |  | |  |  |  |  | |  |
| Netherlands | 1577 | 1 |  |  |  |  |  | |  |  |  |  | |  |
| Sweden | 2040 | 1.94 | (1.59-2.35) | **<0.000*** | N.A | N.A | N.A | | N.A | N.A | N.A | N.A | | N.A |
|  |  |  |  |  |  |  |  | |  |  |  |  | |  |
|  |  |  |  |  |  |  |  | |  |  |  |  | |  |

Abbreviations: OR = odds ratio; CI = confidence interval; ^a^ORs are adjusted for age, sex, educational level, occupation, marital status, drinking categories, healthcare visits in the past 12 months, and country; * = significant at P-value ≤.05. Interaction between predictors and country were non-significant and removed from the model = country*sex: P=0.406; country*age: P=0.958; country*occupation: P=0.100; country*education: P=0.765; country*civil status: P=0.425; country*healthcare visits: P=0.192; country*drinking categories: P=0.571.

**Table 6: Logistic regression model of having had a conversation about alcohol in healthcare in the past 12 months and having reported a positive effect in function of determinants**

|  |  |  |  |  |  |  | |  |  |  |  | |  |  |
| --- | --- | --- | --- | --- | --- | --- | --- | --- | --- | --- | --- | --- | --- | --- |
| **Variables** | **Overall model Sweden Netherlands** | | | | | | | | | | | | |  |
|  |  |  |  |  |  |  |  | |  |  |  |  | |  |
|  | **N** | **OR^a^** | **95%CI** | **p-value** | **N** | **OR^a^** | **95%CI** | | **p-value** | **N** | **OR^a^** | **95%CI** | | **p-value** |
|  |  |  |  |  |  |  |  | |  |  |  |  | |  |
| **Sex**  **Sex** |  |  |  |  |  |  |  | |  |  |  |  | |  |
| Male | 310 | 1 |  |  | 183 | 1 |  | |  | 127 | 1 |  | |  |
| Female | 376 | 0.41 | (0.28-0.59) | **<0.000*** | 240 | 0.41 | (0.24-0.69) | | **<0.001*** | 136 | 0.33 | (0.18-0.59) | | **<0.001*** |
|  |  |  |  |  |  |  |  | |  |  |  |  | |  |
| **Age** |  |  |  |  |  |  |  | |  |  |  |  | |  |
| 16-29 years | 127 | 1 |  |  | 101 | 1 |  | |  | 26 | 1 |  | |  |
| 30-39 years | 128 | 0.70 | (0.37-1.33) | 0.279 | 102 | 0.58 | (0.28-1.19) | | 0.138 | 27 | 1.07 | (0.20-5.60) | | 0.937 |
| 40-49 years | 105 | 0.38 | (0.18-0.79) | **0.009*** | 81 | 0.35 | (0.15-0.79) | | **0.012*** | 24 | 0.37 | (0.06-2.43) | | 0.304 |
| 50-59 years | 120 | 0.62 | (0.32-1.21) | 0.163 | 78 | 0.36 | (0.16-0.81) | | **0.014*** | 42 | 1.63 | (0.34-7.79) | | 0.542 |
| 60+ years | 206 | 0.78 | (0.39-1.56) | 0.485 | 62 | 0.29 | (0.11-0.76) | | **0.012*** | 144 | 2.47 | (0.52-11.76) | | 0.256 |
|  |  |  |  |  |  |  |  | |  |  |  |  | |  |
| **Education** |  |  |  |  |  |  |  | |  |  |  |  | |  |
| Primary education | 35 | 1 |  |  | 21 | 1 |  | |  | 14 | 1 |  | |  |
| Secondary school | 281 | 1.15 | (0.52-2.52) | 0.734 | 197 | 0.86 | (0.30-2.50) | | 0.783 | 84 | 1.50 | (0.42-5.31) | | 0.529 |
| University  Other | 362  8 | 0.72 | (0.32-1.59) | 0.412  0.642 | 205 | 0.68 | (0.23-2.00) | | 0.490 | 157 | 0.72 | (0.21-2.48) | | 0.605 |
| Other | 8 | 0.69 | (0.12-3.99) | 0.680 | N.A | N.A | N.A | | N.A | 8 | 0.49 | (0.05-4.61) | | 0.535 |
|  |  |  |  |  |  |  |  | |  |  |  |  | |  |
| **Occupation** |  |  |  |  |  |  |  | |  |  |  |  | |  |
| Employed | 387 | 1 |  |  | 289 | 1 |  | |  | 98 | 1 |  | |  |
| Student | 57 | 0.78 | (0.36-1.68) | 0.504 | 43 | 0.74 | (0.32-1.75) | | 0.497 | 14 | 0.73 | (0.11-4.77) | | 0.739 |
| Unemployed | 28 | 0.97 | (0.39-2.44) | 0.969 | 22 | 0.62 | (0.20-1.93) | | 0.408 | 6 | 2.65 | (0.29-24.29) | | 0.390 |
| Sick-listed | 40 | 0.73 | (0.32-1.67) | 0.528 | 21 | 0.30 | (0.06-1.43) | | 0.132 | 19 | 0.93 | (0.27-3.24) | | 0.915 |
| Retired | 110 | 1.15 | (0.62-2.13) | 0.555 | 22 | 1.57 | (0.50-4.89) | | 0.438 | 88 | 0.74 | (0.30-1.84) | | 0.521 |
| Other | 64 | 0.74 | (0.37-1.48) | 0.439 | 26 | 0.45 | (0.12-1.65) | | 0.228 | 38 | 0.62 | (0.23-1.65) | | 0.337 |
|  |  |  |  |  |  |  |  | |  |  |  |  | |  |
| **Marital status** |  |  |  |  |  |  |  | |  |  |  |  | |  |
| Married/living together | 452 | 1 |  |  | 281 | 1 |  | |  | 171 | 1 |  | |  |
| Single or living apart | 226 | 1.02 | (0.70-1.49) | 0.911 | 142 | 1.01 | (0.60-1.70) | | 0.962 | 84 | 0.99 | (0.55-1.81) | | 0.989 |
| Other | 8 | 0.47 | (0.10-2.22) | 0.343 | 0 | N.A | N.A | | N.A | 8 | 0.47 | (0.09-2.48) | | 0.375 |
|  |  |  |  |  |  |  |  | |  |  |  |  | |  |
| **Drinking categories** |  |  |  |  |  |  |  | |  |  |  |  | |  |
| **(Adjusted grams=12) grams=12** |  |  |  |  |  |  |  | |  |  |  |  | |  |
| Abstainers | 80 | 1 |  |  | 46 | 1 |  | |  | 34 | 1 |  | |  |
| Moderate drinkers | 453 | 0.83 | (0.47-1.48) | 0.537 | 265 | 1.17 | (0.49-2.79) | | 0.725 | 188 | 0.74 | (0.32-1.72) | | 0.478 |
| Risky drinkers | 153 | 1.73 | (0.91-3.27) | 0.093 | 112 | 2.35 | (0.94-5.88) | | 0.067 | 41 | 1.46 | (0.52-4.07) | | 0.468 |
|  |  |  |  |  |  |  |  | |  |  |  |  | |  |
| **Healthcare visits** |  |  |  |  |  |  |  | |  |  |  |  | |  |
| 1 visit | 157 | 1 |  |  | 122 | 1 |  | |  | 35 | 1 |  | |  |
| 2 or more visits | 529 | 1.15 | (0.74-1.79) | 0.540 | 301 | 1.27 | (0.73-2.20) | | 0.398 | 228 | 1.35 | (0.59-3.11) | | 0.478 |
|  |  |  |  |  |  |  |  | |  |  |  |  | |  |
| **Country** |  |  |  |  |  |  |  | |  |  |  |  | |  |
| Netherlands | 263 | 1 |  |  | N.A |  |  | |  | N.A |  |  | |  |
| Sweden | 423 | 0.37 | (2.24-0.57) | **<0.000*** | N.A | N.A | N.A | | N.A | N.A | N.A | N.A | | N.A |
|  |  |  |  |  |  |  |  | |  |  |  |  | |  |
|  |  |  |  |  |  |  |  | |  |  |  |  | |  |
|  |  |  |  |  |  |  |  | |  |  |  |  | |  |
|  |  |  |  |  |  |  |  | |  |  |  |  | |  |

Abbreviations: OR = odds ratio; CI = confidence interval; ^a^ORs are adjusted for age, sex, educational level, occupation, marital status, drinking categories, healthcare visits in the past 12 months, and country; * = significant at P-value ≤.05, N.A: not applicable. Interactions between predictors and country were non-significant and removed from the model = country*sex: P=0.580; country*age: P=0.064; country*occupation: P=0.528; country*education: P=0.442; country*civil status: P=0.967; country*healthcare visits: P=0.899; country*drinking categories: P=0.745.
